# P-1934. Incidence of Neurotoxicity Among Patients with Leukemia Receiving Vincristine and Azole Prophylaxis

**DOI:** 10.1093/ofid/ofaf695.2102

**Published:** 2026-01-11

**Authors:** Caitlin R Rausch, Kayleigh Marx, Ying Jiang, Elias Jabbour, Dimitrios P Kontoyiannis

**Affiliations:** The University of Texas MD Anderson Cancer Center, Houston, TX; The University of Texas MD Anderson Cancer Center, Houston, TX; The University of Texas MD Anderson Cancer Center, Houston, TX; University of Texas MD Anderson Cancer Center, Houston, Texas; The University of Texas MD Anderson Cancer Center, Houston, TX

## Abstract

**Background:**

Vincristine (VCR) is an essential component of combination chemotherapy for patients (pts) with acute lymphoblastic leukemia (ALL). VCR neurotoxicity (NTX) which can present as peripheral neuropathy, constipation, or ileus, is a well-known dose-limiting side effect. As pts with ALL are at risk of invasive fungal infections (IFIs), azoles are often used as prophylaxis, but excess NTX is a concern due to the inhibition of VCR metabolism. Our practice is to hold the azole for 72 hours around VCR administration. There is limited data on the clinical significance of the azole-VCR interaction in the adult pts with ALL.

**Methods:**

We reviewed the records of all pts > 18 yrs with newly diagnosed ALL who received treatment with Hyper-CVAD (hyperfractionated cyclophosphamide, vincristine, doxorubicin, dexamethasone) with ofatumumab (O-Hyper-CVAD; NCT01363128) or ponatinib (NCT01424982). NTX was graded according to CTCAE v5.0 and IFIs classified according to EORTC/MSG criteria.

**Results:**

A total of 289 VCR episodes were evaluated among 111 pts. 81 pts (73%) received azole prophylaxis, mostly voriconazole ( VOR; 53%). Forty-one pts (37%) experienced NTX. Median age, frequency of concomitant neurotoxic agents, and baseline neuropathy, diabetes, and liver function tests were similar between pts with or without NTX. Twenty-six pts (63%) who experienced NTX received O-Hyper-CVAD and 15 pts (37%) Hyper-CVAD and ponatinib (p=0.07). Median time between VCR initiation and NTX onset was 54 days (range, 1-183). NTX was typically grade 1 (54%) or 2 (39%) and included peripheral neuropathy (85%) and ileus/constipation (20%). Among all VCR episodes, azoles were administered in 58% of those with NTX reported and in 48% of those without (p=NS). VOR was administered in 50% of the VCR episodes with NTX versus 33% of the episodes without NTX (p=0.043). Concomitant VOR was the only factor significantly associated with an increased risk of NTX (OR: 2.49; 95% CI: 1.22-5.12, p=0.013). Only 2 pts (2%) developed an IFI (1 *Rhizopus* sinusitis; 1 probable pulmonary aspergillosis) while on VOR prophylaxis.

**Conclusion:**

Although severe NTX was uncommon during VCR and azole co-administration, VOR may increase the risk of NTX. The rate of breakthrough IFIs is low in contemporary adult ALL pts who are predominantly on azole prophylaxis.

**Disclosures:**

Elias Jabbour, MD, Abbvie: Grant/Research Support|Adaptive Biotechnologies: Grant/Research Support|Amgen Pharmaceuticals: Grant/Research Support|Ascentage: Grant/Research Support|Novartis: Grant/Research Support|Pfizer: Grant/Research Support|Takeda: Grant/Research Support Dimitrios P. Kontoyiannis, MD, AbbVie, Inc: Advisory Board Participation|Astellas: Grant/Research Support|Basilea: Advisor/Consultant|Cidara, Inc: Advisory Board Participation|F2G, Inc: Advisor/Consultant|Gilead: Advisor/Consultant|Gilead: Grant/Research Support|Knight, Inc: Honoraria|Pfizer: Advisor/Consultant|Scynexis: Advisor/Consultant|TTF Pharmaceuticals, Inc: Advisor/Consultant|US-Israel Binational Sci Foundation: Grant/Research Support

